# Vaginal delivery of the recombinant HIV-1 clade-C trimeric gp140 envelope protein CN54gp140 within novel rheologically structured vehicles elicits specific immune responses

**DOI:** 10.1016/j.vaccine.2009.08.088

**Published:** 2009-11-12

**Authors:** Rhonda M. Curran, Louise Donnelly, Ryan J. Morrow, Carol Fraser, Gavin Andrews, Martin Cranage, R. Karl Malcolm, Robin J. Shattock, A. David Woolfson

**Affiliations:** a The School of Pharmacy, The Queen's University of Belfast, Medical Biology Centre, 97 Lisburn Road, Belfast, BT9 7BL, Northern Ireland, United Kingdom; b Centre for Infection, Division of Cellular & Molecular Medicine, St. George's University of London, London, SW17 0RE, United Kingdom

**Keywords:** Vaginal delivery, Mucosal vaccination, HIV-1

## Abstract

Rheologically structured vehicle (RSV) gels were developed as delivery systems for vaginal mucosal vaccination with an HIV-1 envelope glycoprotein (CN54gp140). RSVs comprised a mucoadhesive matrix-forming and vaginal fluid absorbing polymer. The mucoadhesive and rheological properties of the RSVs were evaluated *in vitro*, and the distribution, antigenicity and release of CN54gp140 were analysed by ELISA. CN54gp140 was uniformly distributed within the RSVs and continuously released *in vitro* in an antigenically intact form over 24 h. Vaginal administration to rabbits induced specific serum IgG, and IgG and IgA in genital tract secretions. The RSVs are a viable delivery modality for vaginal immunization.

## 1 Introduction

The world is currently in the midst of a global HIV/AIDS pandemic. UNAIDS estimates 33 million people are currently living with HIV/AIDS with 2.7 million newly acquired infections in 2007 alone [1]. The pandemic is fuelled predominantly by heterosexual transmission of the virus [2], and the last decade has been characterised by a disproportionate HIV-1 infection burden in women [3]. Globally, almost half of all HIV infected individuals are women who have acquired the virus by heterosexual exposure [4–6].

It is broadly acknowledged that the ultimate prophylactic strategy for HIV/AIDS would be the development of an efficacious vaccine. However, the mechanisms by which HIV-1 evades antibody-mediated neutralisation have hindered the development of a potent vaccine [7,8]. Over two decades into the HIV pandemic and a vaccine remains as elusive as ever. The pandemic continues to outpace global effort at prevention and control [9]. In the absence of an effective vaccine strategy, HIV prevention has evolved to be a very multilateral term encompassing structural, behavioural and the biomedical preventive strategies, that now include vaginal microbicides, oral pre-exposure prophylaxis, implementation of male circumcision, highly active antiretroviral therapy, and of course vaccine development [10–13].

As such, there remains a profound scientific rationale to pursue and emphasise the development of female-controlled preventive strategies, principally involving the cervix and vagina as the predominant mucosal viral portal of entry in heterosexual transmission, with a view to eliciting sterilising immunity at the primary point of viral entry. Such a vaginal vaccine should be inexpensive, safe, stable, easy to store and should induce long-lasting high-titre protective mucosal and systemic immune responses to diverse viral isolates through repeated and/or sustained female-controlled administration.

In the case of HIV-1 the envelope spike is the only viral target available for neutralising antibodies [14]. As a result, the majority of candidate HIV-1 vaccines have involved viral surface envelope glycoproteins. The therapeutic efficacy of proteins as pharmaceuticals depends largely on maintaining intended biological activity, and in the case of protein vaccines maintaining intended antigenicity. Vaginal vaccination approaches are well documented in the literature; most involve the administration of antigen in simple buffer solutions rather than delivery modalities specifically designed for vaginal administration [15–19].

From a formulation perspective, inducing effective antigen-specific immune responses by cervicovaginal instillation of buffer solution containing solubilised antigen is far from ideal owing to the potential for leakage at the administration site, rapid enzymatic degradation of the antigen, the influence of the menstrual cycle, and inadequate exposure of antigen to the mucosal associated lymphoid tissue. It is considered that administration of simple antigen solutions is unlikely to promote the prolonged mucosal contact required for effective uptake of antigen and immunogenicity at the vaginal mucosal epithelium.

A limited number of alternative approaches for vaginal administration of antigens have been reported, including antigen-soaked tampons [20] or suppositories [21]. However, there is a strong rationale for expanding the formulation options for effective vaginal administration of vaccine candidates. As a result, the true potential of protein pharmaceuticals as vaccines for intravaginal administration is and will be dependent on advances in formulation science that will promote effective stability, delivery, protection and prolonged exposure of antigen to target cells. Potentially, this may be achieved through sustained-release semi-solid polymeric delivery platforms. To date, the relative success of sustained-release polymeric based protein delivery systems has largely been constrained to low-molecular weight peptides [22]. However, semi-solid polymeric delivery platforms, such as thermosensitive in situ gelling mucoadhesive systems developed for the sustained vaginal delivery of large molecular weight recombinant hepatitis B surface antigen (HBsAg) [23] and human papillomavirus 16 L1 virus-like particles (HPV 16 L1 VLP) [24], are continuously being developed to effectively deliver biomacromolecules and have been shown to enhance vaginal immunization. Such innovative approaches are required for the vaginal delivery of relatively large molecular weight HIV-1 envelope proteins.

In this manuscript we describe the development and characterisation of an innovative gel delivery method for vaginal mucosal vaccination with an HIV-1 vaccine candidate, a clade-C recombinant trimeric envelope glycoprotein (CN54gp140). Vaginal administration of these polymeric, highly viscous, mucoadhesive, retentive, gels (so-called rheologically structured vehicles, RSVs) was evaluated in a rabbit model. The RSV gels in this study contain multiple polymer components, including a mucoadhesive and hydrophilic matrix-forming polymer and a polymeric component capable of absorbing vaginal fluid and potentially offering additional retention advantages.

## 2 Materials and methods

### 2.1 Materials

Hydroxyethylcellulose (HEC/Natrosol 250-HHX-Pharm), Hydroxyethylcellulose (HEC/Natrosol 250-HX-Pharm) and Polycarbophil (Noveon AA1) were kindly donated by Aqualon (Warrington, UK) and Noveon Pharma GmbH & Co KG (Raubling, Germany). (Poly)vinylpyrollidone (Plasdone K-90) was kindly donated by International Speciality Products (Ohio, USA). Galanthus nivalis (GNA) was obtained from Vector Laboratories (Peterborough, England). 3,3′,5,5′-Tetramethylbenzidine peroxidase substrate (TMB/E) was obtained from Millipore U.K. Ltd. (Watford, England). The HIV-1 gp41 specific monoclonal antibody 5F3 utilized for ELISA was obtained from Polymun Scientific (Vienna, Austria). GMP manufactured Carbopol^®^ 974P gel, formulation #2449 was kindly donated by Particle Sciences (Bethlehem, PA, USA). All other chemicals were purchased from Sigma–Aldrich (Poole, Dorset, UK) and were of AnalaR, or equivalent quality. Reactibind 96 well microplates were obtained from Perbio Science (Northumberland, England).

The recombinant trimeric envelope glycoprotein CN54gp140 was supplied by S. Jeffs (Imperial College, London). CN54gp140 was encoded by the CN54gp140REKR HIV-1 envelope gene cassette derived from the clade-C/B’ HIV-1 molecular clone p97CN54 of Chinese origin developed by Wolf and Wagner, University of Regensburg, Germany [25]. The protein was expressed in CHO cells following removal of the membrane-spanning and intracytoplasmic domains of gp41. CN54gp140 was also manufactured to GMP specification and supplied by Polymun Scientific (Vienna, Austria).

### 2.2 Preparation of gel formulations

The RSV gel formulations were hand-mixed in a step-wise fashion following thorough mixing under vacuum using the HiVac™ Bowl bone cement mixing system (Summit Medical Limited, Gloucestershire, UK). A 3% (w/w) Noveon AA1 gel with 0.1% (w/w) sorbic acid in sterile water was prepared and pH adjusted to pH 6 using sodium hydroxide. HEC/Natrosol 250-HHX-Pharm was added at 3% (3% RSV) or 5% (5% RSV) (w/w) and Plasdone K-90 was added at 4% (w/w). The RSV gel formulations for the *in vitro* studies were centrifuged for two periods of 30 min at 3000 rpm (Jouan C312).

HEC gel was prepared based on the recipe of the “Universal Placebo” gel [26] and was prepared by dissolving 1.1% benzyl alcohol, 0.85% sodium chloride, 0.01% sodium hydroxide in 95.16% (w/w) deionised water; followed by slowly dissolving 2.7% Natrosol 250-HX-Pharm using an overhead motorized stirrer with a propeller blade (Heidolph, Schwabach, Germany). The final pH was determined and adjusted if necessary to pH 7.

### 2.3 Measurement of the force required to expel the RSV gel formulations from a syringe

“Syringeability” was determined by measuring the work required to expel the RSV gel formulations from a syringe, using the texture analyser (Stable Micro Systems) with texture profile analysis probe (TPA) in compression mode. To measure the ease of delivery of the RSV gels, 3 g was packed into a modified syringe (tip and base removed), whilst minimising the introduction of air. The syringe was then vertically clamped and the TPA probe was lowered until initial contact with the syringe plunger was observed. The probe was lowered at a rate of 2.0 mm/s through a distance of 30 mm and the resistance to expression of the syringe contents (work done) was determined from the area under the force–time plot recorded during compression of the plunger.

### 2.4 Evaluation of the mucoadhesive strength of the RSV gel formulations

Mucoadhesive strength was determined using the texture analyser in tension mode, to measure the force required to detach a mucin disc from the surface of the RSV gels. Porcine mucin discs (250 mg) were prepared by compression in a Carver press (13 mm diameter die) for 30 s using a defined compression force (10 tonnes) and horizontally attached to the bottom end of a TPA probe using sticky fixers. Immediately prior to mucoadhesive testing, the disc was hydrated by immersion in a 5% mucin solution for 30 s. RSV gel samples packed into shallow cylindrical vessels were placed under the probe which was lowered until the attached hydrated mucin disc contacted the RSV gel surface. A force of 1 N was then applied for 30 s ensuring intimate contact between the disc and the RSV gel. The force required to detach the mucin disc from the sample was then determined by moving the probe upward at a rate of 1.0 mm/s and is defined as the peak value of the resultant force-time plot.

### 2.5 Rheological analysis of RSV gel formulations

Rheological properties can to an extent define the predicted behaviour of a material *in vivo*. Oscillatory data permits interpretation of how the RSVs will behave under low stresses or strain, how their characteristic will effect the release properties and passive seepage over epithelial tissues. Comparison of structural data obtained from rheological evaluation of the RSVs in this study may provide useful information for the prediction of clinical performance. Oscillatory rheological analyses were performed using an AR2000 rheometer (T.A. Instruments, Surrey, England) at 25 °C with a 2 cm diameter parallel plate geometry and a plate gap of 1000 μm. Samples were applied to the upper plate using a spatula to avoid shearing and were applied to the Plate 5 min before analysis to allow for the relaxation of stresses introduced during transferral. Analyses were performed in at least triplicate over a frequency range from 0.1 to 10 Hz. The linear viscoelastic region (LVR) was investigated by increasing oscillatory stress (torque sweep between 1 and 30 Pa) at a fixed frequency (0.1 and 10 Hz) and was identified as the region where the stress and strain were directly proportional and the storage modulus (*G*′) remained constant. Calculation of *G*′, loss modulus (*G*′′) and dynamic viscosity were performed using proprietary software.

### 2.6 Point-of-use syringe mixing

Two 5 ml syringes were attached via a 1.5 cm length of polypropylene tubing with an internal diameter of 3 mm. A solution of a model water-soluble compound (erioglaucine) or of CN54gp140 was added to an aliquot of 5% RSV or 3% RSV respectively, in the same barrel of one of the 5 ml syringes to a final volume of 3 g. Erioglaucine or CN54gp140 and RSV were combined together by 40 passes from one syringe to another via the polypropylene tubing.

The point-of-use syringe mixing method was validated with erioglaucine to preserve stock CN54gp140. Five aliquots of erioglaucine loaded syringe mixed 5% RSV (expected concentration 15.8 μg/0.25 g 5% RSV) were diluted overnight in 100 ml deionised water in an orbital incubated shaker at 37 ˚C. The concentration of erioglaucine was analysed by UV spectrophotometry (630 nm).

### 2.7 Construction of CN54gp140 spiked RSV gel calibration curves for ELISA

Two calibration curve formats were trialled for construction of a “CN54gp140-spiked 3% RSV” calibration curve that could correct for potential matrix effects and loss of detectable CN54gp140 post-formulation and overnight sample preparation. Format 1 involved the individual preparation of each point of the calibration curve by loading 3% RSV gels with different CN54gp140 concentrations and diluting overnight in 0.01 M phosphate buffer (pH 7.4) containing 0.05% Tween-20 and 0.14 M NaCl (PBST) in an orbital incubated shaker (Infors HT) at 37 °C and 60 rpm. Format 2 involved the preparation of a CN54gp140 loaded 3% RSV gel as the top point of the calibration and a blank 3% RSV gel which was used following overnight incubation in PBST to dilute the CN54gp140 loaded RSV gel for construction of the calibration curve. The lower limits of detection (LOD) were calculated using 3 SD of each blank sample.

### 2.8 ELISA for the detection/quantification of CN54gp140

A heterogeneous indirect sandwich ELISA was optimised for intended end-use, to detect and quantify CN54gp140 in diluted RSV gels. Wells were incubated with 50 μl/well of GNA at 10 μg/ml in deionised water overnight at 37 °C. The wells were washed (wash procedure was 5 washes in PBST) and blocked for 1 h at ambient temperature with PBST-serum (PBST with 5% sterile-filtered porcine serum). Standards, samples and controls were diluted in PBST-serum and incubated for 2 h at ambient temperature. The wells were washed and incubated with 50 μl/well HuMab 5F3 for 1 h at ambient temperature. The wells were washed and bound antibody was detected using goat anti-human immunoglobulin (IgG) peroxidase conjugate diluted 1:5 K in PBST-serum and incubated for 1 h at ambient temperature. After washing, the wells were incubated with 100 μl TMB/E. The reaction was terminated by the addition of 50 μl of 2.5 M H_2_SO_4_. The corrected mean of the quadruple absorbance (*A*_450_) measurements of each sample was obtained and compared with those of the negative controls.

### 2.9 Uniformity of mixing

Uniformity of mixing of formulating CN54gp140 within the 3% RSV by the syringe mixing procedure was analysed using the ELISA method. Five single dose 3% RSVs containing CN54gp140 (106 μg per 3 g gel) were prepared using the syringe mixing procedure. 0.5 g aliquots of the single dose 3% RSVs were weighed into 100 ml sterile screw-cap polypropylene containers and diluted in 100 ml PBST overnight in an orbital incubator at 37 °C and 60 rpm. The concentration of CN54gp140 in each aliquot was determined by ELISA.

### 2.10 *In vitro* CN54gp140 release studies

#### 2.10.1 Cap method

Five single dose 3% RSV formulations were prepared to a CN54gp140 loading of 100 μg per 3 g 3% RSV and transferred to the inside of a McCartney vial cap. The McCartney vial caps were fixed to the bottom of 100 ml sterile screw-cap polypropylene containers using vacuum grease. The McCartney vial caps containing CN54gp140 loaded 3% RSV gel were immersed in 30 ml PBST and maintained at 37 °C and stirred at 60 rpm in an orbital incubator. At designated time intervals 3 ml of release media was removed for analysis and replaced with 3 ml of fresh PBST. When it was necessary release samples were stored at 4 °C before analysis by ELISA.

#### 2.10.2 Expulsion method

The expulsion release method was as per the cap method with the exception that CN54gp140 loaded gels (100 μg/3 g 3% RSV; 98 μg/3 g HEC; 98 μg/3 g Carbopol^®^) were expulsed into the release media as opposed to being contained within McCartney vial caps.

### 2.11 Assessment of the stability of CN54gp140 formulated within the RSV gel

Three single dose 3% RSVs containing CN54gp140 (106 μg per 3 g gel) were prepared using the syringe mixing procedure. The recovery of CN54gp140 from 0.5 g aliquots of the single dose 3% RSVs stored at three different temperatures (4 °C, ambient, 37 °C) was monitored over time. Following remixing of the CN54gp140 loaded 3% RSV gel at each time point the aliquots were weighed into 100 ml sterile screw-cap polypropylene containers and diluted in 100 ml PBST overnight in an orbital incubator at 37 ˚C and 60 rpm. The concentration of CN54gp140 in each aliquot was determined by ELISA.

### 2.12 Immunogenicity/toxicology-irritancy study

#### 2.12.1 *In vivo* procedures

12 female 10–12-week-old New Zealand white rabbits were each given 9 intravaginal immunizations of 65 μg of CN54gp140 in either of two RSV gel formulations: 3% RSV or 5% RSV, at a total volume of 400 μl administered at days 1, 3, 5, 8, 10, 12, 15, 17 and 19. Just prior to administration, antigen and gel were mixed according to the point-of-use syringe mixing method. Air was removed from each syringe by centrifugation at 400 × *g* and the homogenous mixture was transferred to 1 ml syringes for dosing, to allow for accurate administration. The gel formulations were introduced approximately 6 cm into the vagina via a soft rubber catheter attached to the syringe. Blood samples for serological, haematological and blood chemistry analysis were taken pre-treatment and on days 6, 13 and 20. Secretions from the vagina and the vestibule were sampled using ophthalmic sponges (Weck-Cel™ spears, Medtronic Xomed, Inc.) *post mortem* from tissues that had been removed for this specific purpose (so as not to interfere with histological analysis). A sterile spear was placed on the mucosal surface of the vagina for 2 min to absorb any secretion. A second spear was placed on the mucosal surface of the vestibule. Secretions were eluted from the sponges by placing each spear into a spin-X tube (Costar) containing 300 μl extraction buffer (protease inhibitor cocktail set I, Calbiochem, EMD Biosciences). Samples were frozen at −80 °C before dispatch to the laboratory. Standardised procedures were used for the assessment of any toxicological or irritancy effects including visual inspection at least twice daily for evidence of reaction to treatment or ill health and weekly physical examination. Animals were observed for signs of vulval irritation, discharge or bleeding from the vagina before each intravaginal administration and any indications were graded on a standardised irritancy scale. Body weights were recorded one week before study commencement and regularly then at the first and following each subsequent intravaginal inoculation. Animals were killed humanely at day 20 and detailed macroscopic and histological analysis undertaken. All *in vivo* and necropsy procedures were carried out by a Contract Research Organisation on behalf of the sponsors, as part of a preclinical evaluation for Phase I testing in humans. All *in vivo* procedures were carried out in compliance with the United Kingdom Animals (Scientific Procedures) Act 1986 and animals were maintained in compliance with the associated Codes of Practice for the Housing and Care of Animals.

#### 2.12.2 Assay of antibody responses

IgG binding antibodies were measured using a standardised ELISA. 96-well plates (Greiner Bio-One medium binding) were coated with CN54gp140 at 5 μg/ml in phosphate buffered saline (PBS) for 1 h at 37 °C. Plates were washed 4 times in PBST before blocking with PBST containing 10% foetal bovine serum for 1 h at 37 °C. After further washing, sera diluted in PBST or mucosal eluates were added and incubated for 1 h at 37 °C. Bound antibody was detected with monoclonal anti-rabbit IgG (γ-specific) peroxidase conjugate (Sigma A1949) diluted to optimal concentration as determined by checkerboard titration. After incubation and washing as above, colour was developed by addition of TMB (Sureblue TMB 1-component peroxidase substrate (KPL)). The reaction was stopped with TMB Stop Solution (KPL) after 5 min incubation in the dark and the plates were read immediately at 450 nm.

For the detection of IgA antibody a modified ELISA protocol was used that had previously been optimised for sensitivity and discrimination of positive and negative control samples, based on a human immunoglobulin ELISA. Bound IgA was detected by the addition of biotin conjugated to goat anti-rabbit IgA (α-specific) (Autogenbioclear ABN116B) followed by avidin conjugated to horseradish peroxidase (Sigma A7419). The substrate reaction was allowed to proceed for 10 min for the IgA ELISA. Standard curves for IgG detection were derived using rabbit antiserum to HIV-1 GB8 gp120 (Programme EVA, Centre for AIDS Reagents, National Institute for Biological Standards and Control, ARP440.1/R546). A specific IgA antibody containing rabbit serum was not available. All assays used normal rabbit serum (Sigma, R9133) as a negative control. For initial screening, serum samples were tested in triplicate at 1:100 and mucosal samples were tested undiluted. An absorbance greater than or equal to 0.2 was used as the positive cut-off.

## 3 Results

### 3.1 Rheological analysis, mucoadhesive strength and syringeability

The 3% RSV gel formulation was compared to the 5% RSV gel formulation. The change in rheological viscoelastic properties (*G*′, *G*″, and *η*′ at 5 representative frequencies) and mechanical properties associated with the inclusion of different percentages of HEC were determined by oscillatory rheology and TPA respectively (Table 1).

5% RSV was more rheologically structured than 3% RSV displaying higher values of *G*′, *G*″ and *η*′ (across the entire frequency range studied), parameters which signify how elastic, liquid and viscous a material is. The values of *G*′ and *G*″ increased as a function of frequency. Physically entangled networks characteristically display increases in *G*′ and *G*″ with increasing oscillatory frequency, however the increases observed here were relatively small suggesting a high degree of physical interaction between the polymeric components [27].

The lower values of *η*′ observed with the 3% RSV would suggest that this lower viscosity formulation may be more easily applied than the 5% RSV formulation, using a vaginal gel applicator, and in turn may more readily coat the epithelial surfaces. The lower hardness and compressability values obtained during TPA studies and the lower amount of work required to expel the formulation in syringeablility studies (employing a device resembling that typically used as a vaginal gel applicator) in comparison to 5% RSV corroborate this. Despite ease of administration, higher levels of mucoadhesiveness were maintained and are likely to ensure prolonged adhesion of the formulation to the mucosal surfaces.

### 3.2 Validation of the syringe mixing method

Assuming homogeneous distribution of erioglaucine post-syringe mixing, a 0.25 g aliquot of 5% RSV had an expected erioglaucine loading of 15.8 μg. Theoretical concentration of dispersed erioglaucine aliquots was 158 ng/ml. The mean actual concentration as determined by UV spectrophotometry was 147.9 ± 6.2 ng/ml (RSD 4.2) indicating homogeneous distribution of erioglaucine throughout the 5% RSV.

### 3.3 ELISA for the detection/quantification of CN54gp140

**Fig. 1:**
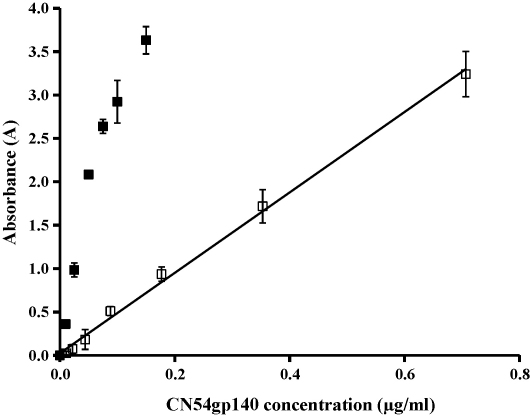
Calibration curves for the indirect heterogeneous sandwich ELISA of free-CN54gp140 (□ CN54gp140-spiked 3% RSV, ■ CN54gp140 in PBST, *n* = 4).

No difference was observed between Format 1 and 2 for the construction of CN54gp140-spiked 3% RSV calibration curves, therefore format 2 was selected to ensure minimal use of CN54gp140. A linear calibration plot for CN54gp140 in PBST was observed over the range 0.003–0.05 μg/ml (*R*^2^ 0.9988) and for the CN54gp140-spiked 3% RSV curve over the range 0.01–0.7 μg/ml (*R*^2^ 0.9968) (Fig. 1). The LOD of CN54gp140 in PBST was 0.003 μg/ml and the RSD over the entire linear range of the assay of was <8.1%. The LOD of CN54gp140-spiked 3% RSV was 0.01 μg/ml and the RSD was <66% until 0.04 μg/ml (4LOD) and <11% thereafter. The *A*_450_ values of the linear range of CN54gp140 in PBST assay were on average 92% higher than those of the CN54gp140-spiked 3% RSV assay. When required samples were diluted to within the linear range of the assay. A CN54gp140-spiked 3% RSV curve was applied to the uniformity of mixing study and stability study and a CN54gp140 in PBST curve was applied to the release studies.

### 3.4 Uniformity of mixing

Assuming homogeneous distribution of CN54gp140 post-syringe mixing, a 0.5 g aliquot of 3% RSV had an expected CN54gp140 loading of 17.6 μg. Theoretical concentration of CN54gp140 in diluted RSV gel was 0.35 μg/ml. The mean actual concentration as determined by ELISA was 0.36 ± 0.02 μg/ml (RSD 5.3).

### 3.5 *In vitro* release data for CN54gp140

**Fig. 2:**
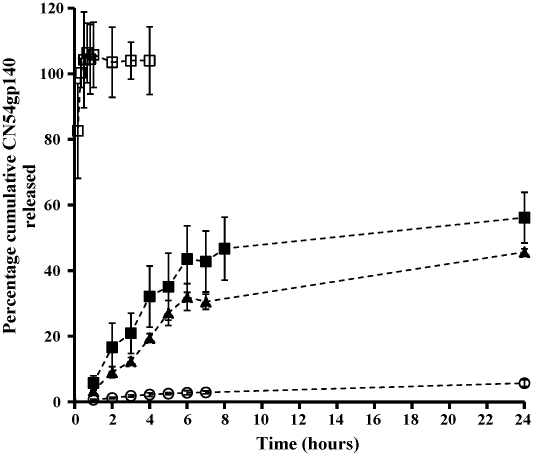
Mean percentage cumulative release versus time profile for CN54gp140 (○ confined 3% RSV, *n* = 5, ▴ expulsed 3% RSV, *n* = 5, ■ expulsed HEC, *n* = 5, □ expulsed Carbopol^®^, *n* = 3.

The degree of matrix associated dampening was expected to vary over the course of the release studies therefore the concentrations of CN54gp140 were determined using CN54gp140 in PBST calibration curves and the data interpreted as minimum release. The percentage cumulative release profiles of CN54gp140 from 3% RSV gels (cap method: RSD 0.15–24.70, expulsion method: RSD 2.11–18.42), from HEC gels (RSD 3.72–44.15) and from Carbopol^®^ gels (RSD 0.88–17.85) are shown in Fig. 2. Using the expulsion method 45.66% of CN54gp140 was released within 24 h, 8-fold higher than when using the cap method (5.70%). Using the expulsion method 45.7% of CN54gp140 was released from the 3% RSV gel and 57.36% from the HEC gel within 24 h, compared to 98.17% within 20 min in the case of the Carbopol^®^ gel (Fig. 2).

### 3.6 Stability data

Recovery values of CN54gp140 from 0.5 g aliquots of 3% RSV at different stability storage temperatures were as follows: +4 °C—92% Day 2, 92% Day 3 and 81% Day 9; *ambient—*83% Day 2, 82% Day 3, 74% Day 4, 59% Day 9; 37 °C—77% Day 2, 53% Day 3, 42% Day 4, and 21% Day 9.

### 3.7 Vaginal irritation and toxicity

All animals survived to the end of the treatment period and no signs of systemic toxicity were observed following administration of the vaccine in either of the gel formulations. There were no effects on body weight, food consumption or organ weights and there were no treatment-related findings by macroscopic examination. Changes in the vagina determined by histological examination were scored using the method of D’Cruz et al. [28] modified from that of Eckstein et al. [29]. Overall cumulative scores fell within the acceptable range for human vaginal irritation potential (0–8) for all animals. Animals given CN54gp140 in 3% RSV had the lowest scores, ranging from 1 to 3; whereas, animals receiving antigen formulated in 5% RSV had scores ranging from 4 to 7.

### 3.8 Induction of specific antibody responses

**Fig. 3:**
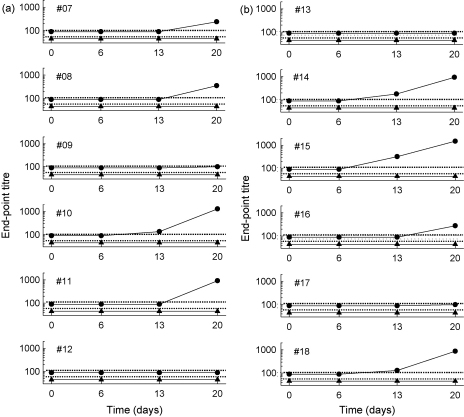
Intravaginal immunization of rabbits with CN54gp140 formulated in 3% RSV (a) or 5% RSV stimulated production of systemic IgG anti-gp140 antibody. IgG (●) and IgA (▴) gp-140-specific ELISA titres were determined by dilution to a standardized end-point and the limits of detection are shown for IgG () and IgA ().

Five of six rabbits receiving CN54gp140 antigen formulated in 3% RSV gel and 5 of 6 animals receiving antigen formulated in 5% RSV gel had evidence of specific antibody responses detectable in serum (Fig. 3) and in secretions from the female genital tract (Table 2). Serum IgG antibody was detected as early as day 13 in 1 of the animals receiving 3% RSV and in 3 of 6 animals receiving 5% RSV with titres ranging from 130 to 320. By day 20, the last day of the study, IgG antibody titres had increased in all these animals and another 6 animals had sero-converted, although in two animals, one from each group, titres only just reached the minimum detectable. Overall, serum IgG antibody titres ranged from 100 to 1500 at day 20. Titres were broadly similar between the two groups (100–1280 and 100–1500 for each group respectively). IgA antibody was undetectable in any serum sample at or above the minimum titre of 10.

Female genital tract secretions from the two animals that failed to sero-convert also tested negative for antibody. All other animals, albeit sometimes at low titre, had evidence of antibody in vaginal and or vestibule secretions. Four animals in each group had IgG specific antibody detectable in mucosal samples with titres that were broadly associated with IgG titres in serum. Interestingly, 4 animals in each group also had mucosal IgA antibodies and in two cases IgA was detectable in the absence of mucosal IgG. Constraints of volume precluded titration of mucosal samples in most cases; however, in one animal where IgG antibody was undetectable in genital tract secretions IgA was present at relatively high titre.

## 4 Discussion

Due to their low cost, ease of manufacture, and precedence of use in the topical administration of drugs, conventional semi-solid gel systems are commonly employed to administer drugs via the vaginal route, mainly for the treatment of vaginal infection, contraception, and hormone replacement therapy. More recently, gel-based formulations are being widely developed for the delivery of HIV microbicides [30–32]. A small number of studies have reported the use of gel systems for the vaginal delivery of vaccines [23,24,33–35]. However, the poor vaginal retention of conventional gel formulations represents a significant challenge for those clinical indications where sustained delivery is predicted to potentially enhance efficacy, such is the case for the vaginal delivery of HIV vaccines.

The development of protein-based HIV vaccine pharmaceuticals (HIV envelope proteins) is particularly challenging due to the fragility and complex structure of the protein molecules involved [36,37], the cervicovaginal administration of which is further subject to many other constraints such as enzymatic degradation. Whilst the recombinant envelope protein used in this study has proven to be exceptionally stable, relative to other HIV-1 recombinant envelope glycoproteins, in general formulation strategies should avoid processes that may have a detrimental effect on the envelope protein in order to preserve stability and subsequent biological activity [38].

Prolonged retention and intimate contact of antigen with the vaginal mucosa are achievable through manipulation of the rheological and mucoadhesive properties of semisolid platforms. We have developed RSV gel formulations that are designed to provide enhanced mucosal retention within the vagina and have the ability to spread and coat the epithelial surface whilst maintaining rheological structure even when subject to the effects of ambulation, seepage between epithelial surfaces, sexual intercourse and dilution with vaginal fluids. A bioadhesive component was included in the formulation design to promote intimate contact of the polymer with the mucosal surfaces. Although thermoreversible gelling systems may offer the advantage of ease of vaginal administration, the RSV gels are expected to offer retention advantages on account of their dilution-resistant characteristics.

Two RSV gel formulations were used in this study, containing 3% and 5% HEC. The lower viscosity version of the formulation, achieved by reducing the matrix-forming polymer component HEC from 5% to 3%, was investigated to facilitate ease of dispensing and application in a clinical setting. Rheological analyses demonstrated that reducing the HEC component reduced the *G*′ (at a frequency of 1.142 Hz) of the RSV gel from 6149 ± 285 Pa (5% RSV) to 3940 ± 285 Pa. As predicted, this decrease in rheological structure correlated with significant decrease in the syringeability of the RSV gel formulation (work done for 3% RSV 42.58 ± 8.9 N mm, compared to 5% RSV 66.94 ± 9.19 N mm). For comparative purposes the *G*′ (at a frequency of 1.142 Hz) of three considerably less viscous commercial gel preparations were determined by oscillatory rheology: Acijel (1402 ± 156 Pa), Metrogel (177 ± 8 Pa), and Zidoval (642 ± 32 Pa). Texture profile analysis also confirmed lower levels of hardness and compressibility (3.169 ± 0.086 N and 3.182 ± 0.089 N s respectively (3% RSV) compared to 6.282 ± 0.2 N and 6.257 ± 0.1095 N s respectively (5% RSV)), with a concomitant increase in mucoadhesive bond strength (0.467 ± 0.072 N (3% RSV) compared to 0.37 ± 0.041 N (5% RSV)). This trend has been observed before [39,40]. Based on these results, the 3% RSV gel formulation is expected to facilitate ease of vaginal administration compared to the 5% RSV, but still offer considerable retention compared to other more conventional vaginal gel products.

Preliminary evaluation of the point-of-use syringe mixing method was validated using the higher viscosity 5% RSV gel formulation using the model compound erioglaucine. Further studies confirmed that the same mixing protocol was suitable for ensuring homogeneous distribution of CN54gp140 in the lower viscosity 3% RSV gel formulation. All subsequent *in vitro* studies were performed with the 3% RSV gel, although both gels were evaluated in the rabbit studies.

The release study using the expulsion format was considered to more closely model *in vivo* administration (or vaginal smearing). High RSDs were anticipated for the expulsion method as the non-confined nature of this release method leads to variable surface area available for dissolution of CN54gp140 into the surrounding release media. In fact, similar variability in release data for both methods was observed, as measured by RSDs (Fig. 2). The *in vitro* release of CN54gp140 from three different gel formulations (3% RSV, HEC gel, and Carbopol^®^ 974P gel) was monitored over a 24 h period. Almost 100% of CN54gp140 was released from the Carbopol^®^ gel within 10 min. The HEC and RSV gel formulations released in a much more sustained manner. As anticipated, the 3% RSV gel formulation released at the slowest rate. This sustained-release behaviour, in combination with the site-retentive characteristics predicted from the rheological and mucoadhesive data, may render the RSV gel formulation most suitable for use in vaginal immunization. In particular, although the correlates of HIV-1 protective immunity remain unknown, we are assuming that persistent local exposure of the antigen at the mucosal surface is essential for induction of effective adaptive immunity.

Although the GMP manufactured CN54gp140 has proven to be exceptionally stable in simple buffer solutions, early indications suggest that stability is compromised when formulated within the 3% RSV gels. This may have implications for temperature storage and distribution of a gel-based HIV vaccine.

This study was designed primarily to confirm the effectiveness of the RSV gel formulations as vaginal delivery modalities for mucosal vaccination. Using a preliminary animal immunogenicity study (designed to assess the toxicity or local irritation of the RSV gel formulations as a preclinical testing requirement) it was determined that specific immune responses could be achieved using the RSV gels as vaginal delivery vehicles for CN54gp140. It was particularly encouraging that although serum IgA antibodies were not detected evidence of IgA as well as IgG antibodies was obtained from the female genital tract secretions. In particular, the detection of IgA antibodies in secretions suggests that these were of local origin as serum IgA was not detected in the same animals: further studies are required to validate this. IgG antibody may also be generated locally; however the female genital tract is thought to be relatively permeable to antibody derived from the circulation [41]. Small volumes of secretions recovered in this study precluded analysis of antibody titre referenced to total immunoglobulin; however, in an associated study with the same protein formulated in Carbopol^®^ gel the proportion of specific antibody detected in mucosal secretions was 8.5–9.0-fold higher compared to serum [42]. The observation of immune response following vaginal vaccination with the RSV gel formulations is encouraging as the vagina is considered to be a poor inductive site for humoral immunity [43]. Ongoing studies will provide comparative data in different species and will assess whether responses to RSV gel formulations are increased following priming via alternative routes (parenteral or intranasal).

CN54gp140 administered in both RSV gel formulations mounted similar immune responses in both rabbit groups. Although *in vitro* release of CN54gp140 was not carried out for 5% RSV, it may be predicted that the CN54gp140 release rate *in vitro* may be marginally faster from 3% RSV compared to 5% RSV. No such assumptions can be made on the elicitation of immune responses *in vivo*. Although the rabbit is a validated model for vaginal irritancy testing, little is known about the rabbit female genital tract as an inductive site for mucosal and systemic immunity.

## 5 Conclusions

This study has described the design and development of a mucoadhesive, syringeable, rheologically structured gel system for site-retentive application of an HIV vaccine candidate to the vagina. The mechanical and mucoadhesive properties, release profile of CN54gp140 and subsequent elicitation of specific immune responses indicate that the RSV gel may have an advantageous role promoting persistent/intimate exposure of CN54gp140 to the mucosal-associated lymphoid tissue of the female genital, thus augmenting and prolonging CN54gp140-specific local immune responses in comparison to more conventional gel formulations.

## Figures and Tables

**Table 1:** Change in rheological viscoelastic properties (*G*′, *G*″, and *η*′ at 5 representative frequencies) and mechanical properties, with increasing HEC concentration within RSV gel formulations determined by oscillatory rheology and TPA respectively.

Formulation	Oscillatory rheology data			Texture profile analysis				
	Freq. (Hz)	*G*′ (Pa)	*G*″ (Pa)	*η*′ (Pa s)	Mucoadhesive strength (N)	Syringeability (N mm)	Hardness (N)	Compressibility (N s)
3% RSV	1.14	3940 ± 285	965 ± 64	134 ± 9	0.467 ± 0.072	42.58 ± 8.90	3.17 ± 0.09	3.18 ± 0.09
	3.23	4618 ± 330	1046 ± 71	52 ± 3				
	5.31	4939 ± 323	1081 ± 69	32 ± 2				
	7.40	5139 ± 283	1101 ± 59	24 ± 1				
	10.00	5337 ± 257	1122 ± 45	18 ± 1				

5% RSV	1.14	6149 ± 130	1432 ± 33	200 ± 5	0.37 ± 0.04	66.94 ± 9.19	6.28 ± 0.2	6.26 ± 0.11
	3.23	7126 ± 150	1465 ± 38	72 ± 2				
	5.31	7578 ± 150	1474 ± 38	44 ± 1				
	7.40	7853 ± 176	1479 ± 38	32 ± 1				
	10.00	8075 ± 294	1475 ± 47	23 ± 1				

**Table 2:** Intravaginal immunization of rabbits with CN54gp140 formulated in 3% RSV or 5% RSV stimulated production of IgG and IgA gp140-specific antibodies detected in vaginal and vestibular sponge eluates taken 20 days after the start of immunization.

Animal no.	RSV (%)	IgG titre (absorbance)		IgA titre (absorbance)	
		Vagina	Vestibule	Vagina	Vestibule
#07		<1	1	<1	<1
#08		<1	3	3	<1
#09	3	<1	<1	<1	+(0.23)
#10		70	55	+(0.5)	<1
#11		125	12	+(0.36)	<1
#12		<1	<1	<1	<1
#13		<1	<1	<1	<1
#14		650	81	<1	<1
#15	5	50	70	+(0.24)	+(0.24)
#16		<1	<1	37	70
#17		7	3	+(0.57)	+(0.71)
#18		75	1	+(0.99)	+(1.74)

End-point titres are shown where sufficient volume was available for analysis. Where titration was not possible, + indicates a result above the pre-defined cut-off in undiluted eluate with the corresponding absorbance value shown in parentheses.
